# Discovery pattern and species number of scale insects (Hemiptera: Coccoidea)

**DOI:** 10.7717/peerj.2526

**Published:** 2016-09-29

**Authors:** Jun Deng, Kunming Li, Cui Chen, Sanan Wu, Xiaolei Huang

**Affiliations:** 1 College of Plant Protection, Fujian Agriculture and Forestry University, Fuzhou, Fujian, China; 2 College of Economics, Fujian Agriculture and Forestry University, Fuzhou, Fujian, China; 3 The Key Laboratory for Silviculture and Conservation of Ministry of Education, Beijing Forestry University, Beijing, China

**Keywords:** Species diversity, Taxonomic effort, Scale insect, Gompertz model

## Abstract

Few investigations have been made of the species description trend of scale insects. The present study reports the discovery pattern and taxonomic efforts for this group based on global species and a literature dataset. In addition, three asymptotic models (Logistic, Gompertz, and Extreme Value) based on a discovery curve were used to predict the species number of scale insects. Our results showed that the species description rate has been changing over time, with certain peaks and valleys in the past 250 years. The mean number of species described per year was 30, with the highest number of 195 described species in 1985. The increasing number of authors and the almost constant proportion of species described by 10% most prolific authors since the 1900s suggested that taxonomic effort has been increasing over time. The Gompertz model with lowest AIC value suggested that there are about 10,450 species of scale insects on Earth, nearly 30% of which remain to be described. Our study offers insights into the discovery pattern of scale insect diversity.

## Introduction

The question of how many species exist on Earth has attracted attention from scientists for more than 250 years (Ødegaard, 2000). Estimating species number is an essential step in understanding the state of biodiversity, and particularly important due to threats of species extinctions (e.g., habitat loss, invasive species, climate change). Estimates of species richness also have important implications for conservation such as setting conservation priorities for species (Joppa, Roberts & Pimm, 2011). However, because of limited sampling or incomplete data for the world’s biodiversity, it is impractical to quantify the number of species directly (Mora et al., 2011). The indirect methods provide available choices for accomplishing this task (Costello & Wilson, 2011). Predictions based on species description rates are commonly used in many studies (Erwin, 1982; Hodkinson & Casson, 1991; Bebber et al., 2007). The estimated number of global species ranges between two and 10 million (Chapman, 2009; Mora et al., 2011; Costello, Wilson & Houlding, 2012), while the total number of described species approximates 1.6 million (Roskov et al., 2015). Many species, especially small invertebrates, have never been discovered or described (Costello, Wilson & Houlding, 2012).

Scale insects, an economically important plant-feeding group including some notorious agricultural pests such as *Ceroplastes rubens*, *C. rusci* and *Coccus hesperidum*, usually have small bodies (Hamon & Williams, 1984; Gill, 1988). Meanwhile, many scale insects live on indetectable parts of plants such as leaf sheath and root. These traits may preclude discovery of scale insect species in practice. The taxonomy of scale insects began with Carl Linnaeus’ 10th edition of the *Systema Naturae* (Linnaeus, 1758). From then on, the number of scale insects has increased from 24 species (Williams, 2007) to 8,197 species described in 23,477 references (García et al., 2015). These species and references provide an opportunity to investigate discovery pattern and process of scale insects over time.

The main aims of this study were to investigate the current state of knowledge of scale insects biodiversity on a global scale and to predict species number in this group. The temporal trend of species description and number of authors were analyzed in order to assess taxonomic efforts for scale insects. An indirect method based on time–species accumulation curve, which has been widely used for various groups including Scarabaeoidea (Cabrero-Sanudo & Lobo, 2003), birds (Bebber et al., 2007), ants (Bebber et al., 2007), Arctiidae (Ferro & Diniz, 2008), Cyanobacteria (Nabout et al., 2013), and mayfly species (Cardoso et al., 2015), was used to estimate total species number of scale insects. In the present study, the discovery time was defined as the year of description of taxa, such descriptions tend to appear in many groups (Bebber et al., 2007).

## Materials and Methods

### Data collection

For the species data set of scale insects, we first collected species data from the 2015 issue of the Catalogue of Life (CoL), which covered all scale insects described from year 1758 to 2004. Considering the CoL data of scale insects came from the scale insect database, ScaleNet (García et al., 2015), we then compiled data for the period between 2005 and 2014 from the ScaleNet. These two parts combined into a final species data set from 1758 to 2014. Due to a time lag between recent publication and being included into species databases, species data for the year 2015 were excluded. Because discovery of extinct species relies very much on fossils, sixteen extinct scale insect families were also excluded from the current analyses. A total of 7,754 species of extant scale insects were included in the final dataset (Table S1). The year of species description and names of the authors who described the species were extracted for further analyses. Although same surnames included in species names may represent different authors, they were usually considered not to significantly affect results of related analysis (Costello, Wilson & Houlding, 2012).

For the literature data set, we surveyed literature using the Zoological Record™ (Thomson Scientific) in a time range of 1864–2014. Search terms (coccoidea or “scale insects”) and (“new speci*” or “new tax*”) were used for TOPIC search. Some entries with titles including terms “Hymenoptera” or “Diptera” or “Lepidoptera” or “Coleoptera” were excluded. Finally, a total of 2,234 entries including information on authors and publication dates were extracted from the Zoological Record™ database.

### Assessment of taxonomic efforts for scale insects

To estimate taxonomic efforts for scale insects, the numbers of described species, papers and authors per year were counted and analyzed. Considering the large number of descriptions by Douglas J. Williams, when the number of species described per author over time was calculated, those species data with only ‘Williams’ as an author were excluded. Then, the species description patterns of five major families (Diaspididae, Pseudococcidae, Coccidae, Eriococcidae and Margarodidae) were analyzed with the aim to compare taxonomic efforts among taxa.

To investigate the long-term trend in taxonomic efforts over time more closely, several further analyses were conducted. First, the cumulative proportion of species and papers were plotted against the cumulative proportion of authors, respectively. Second, the proportion of species described by the 10% most prolific authors each decade was estimated following the method of Costello, Wilson & Houlding (2012). Third, we also estimated the number of authors who described only one species and the number of species described by them. Finally, we analyzed the temporal trend in the numbers of taxonomists who have more than 5, 10 and 15 years of taxonomic experience, respectively. These people were supposed to be full-time taxonomists. The years on first and last published species descriptions were used to determine the length of taxonomic experience.

### Evaluation of potential species number of scale insects

The accumulation curves were used to simulate the trend of species description over time. In view of potential effect of size, spatial scale and state of knowledge of the taxonomic group, three non-linear models including Logistic, Gompertz (Ratkowsky, 1990) and Extreme value (Williams, 1995) were used to predict the total species number of scale insects. The curve fitting was implemented by using Matlab’s Curve Fitting Toolbox (Matlab 2014a, MathWorks Inc.). Akaike information criterion (AIC) (Akaike, 1973) was used to rank the models and select a best model (Nabout et al., 2013). AIC is commonly used in ecology and evolution (Johnson & Omland, 2004), which estimates the Kullback–Leibler information lost by approximating full reality with the fitted model (Burnham & Anderson, 2002).

## Results

**Figure 1:**
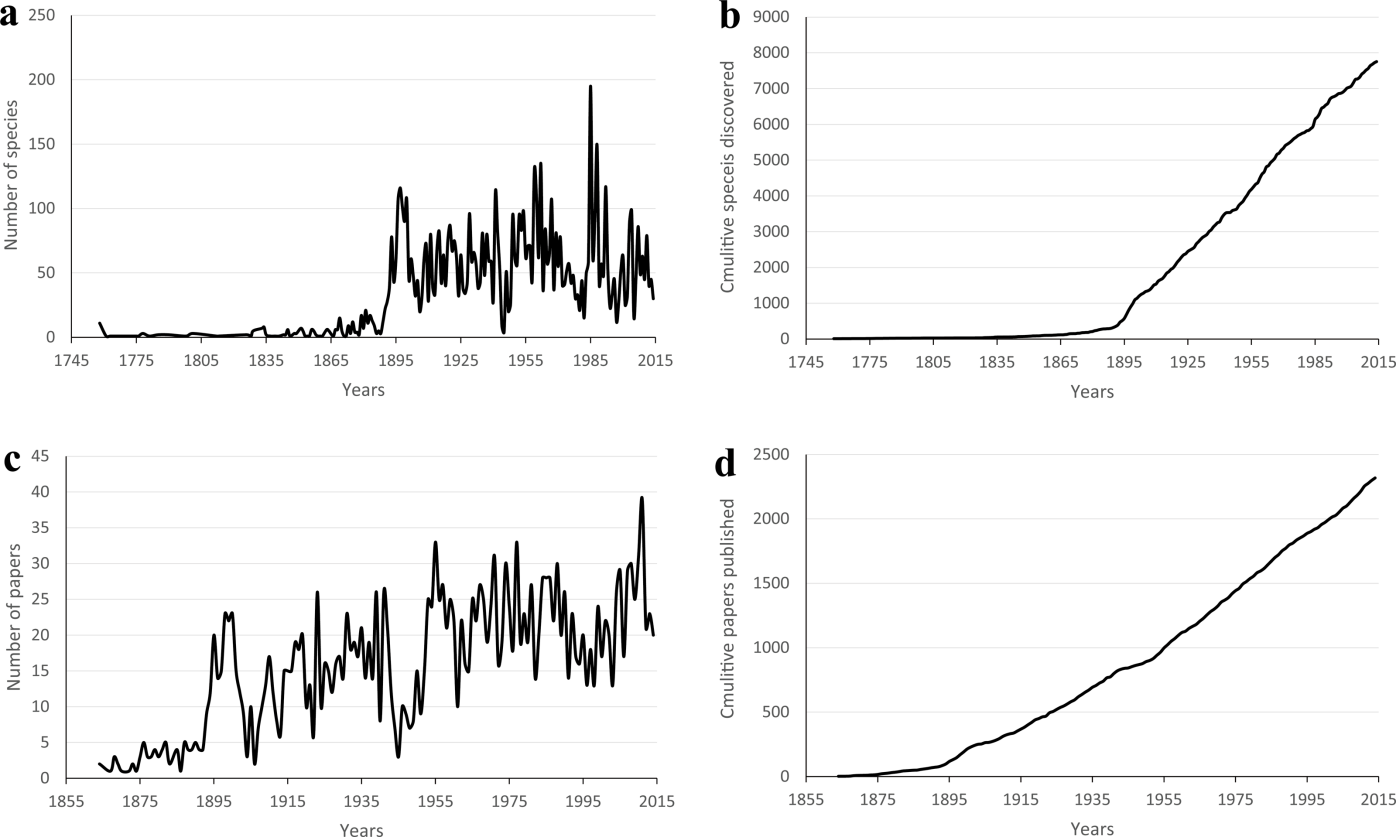
The number of species described and published papers in the CoL and Zoological Record™, respectively. Species number of scale insects described each year (A), cumulative number of scale insects (B), number of published papers each year (C) and cumulative number of published papers (D) from 1758 to 2014.

The analyses of temporal trends of described species of scale insects showed that species description rate has been changing over time, with some peaks and valleys during 1758–2014 (Fig. 1A). The average number of species described per year was 30 (standard deviation 36; 95% confidence interval 26–34), with the highest peak of 195 species in 1985. The species cumulative curve was almost linear since the 1900s (Fig. 1B). For the literature dataset, the average number of published papers per year was 15.4 (SD = 9; 95% CI = 13.9 to 16.9) (Fig. 1C), with the highest value of 39 papers in 2011. The cumulative curve of published papers (Fig. 1D) yielded similar patterns with that of described species.

**Figure 2:**
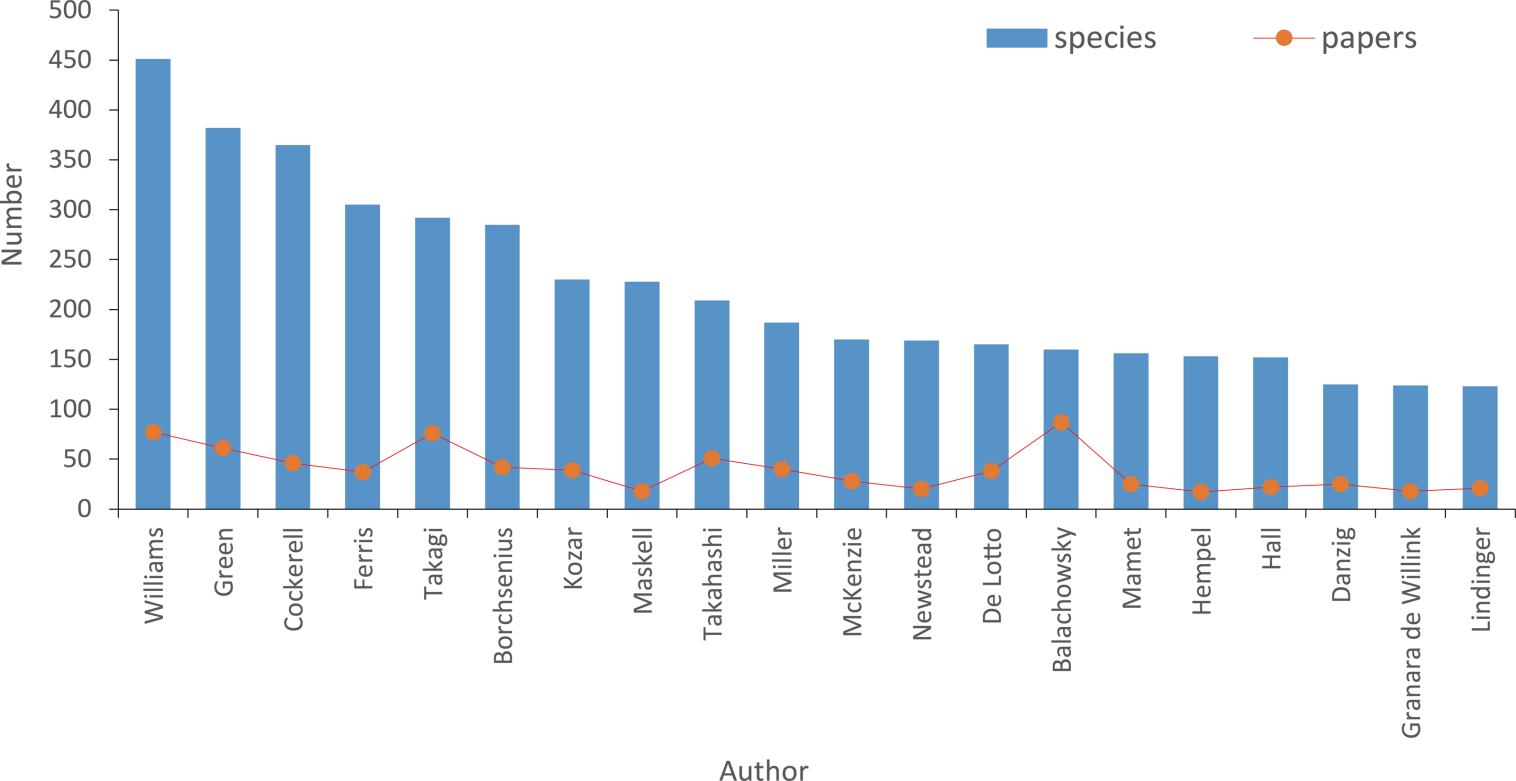
The top 20 authors who described most species of scale insects.

In our data set, a total of 7,754 species of scale insects were described by 538 authors. The top two authors with surnames Williams and Green have named 833 species, representing about 10% of the total species of scale insects. The top 20 authors described more than 50% of scale insects (4,431 species) (Fig. 2). However, productivity in species description did not positively correlate with paper numbers the authors published (Fig. 2). This may be simply due to their difference in publishing behavior.

**Figure 3:**
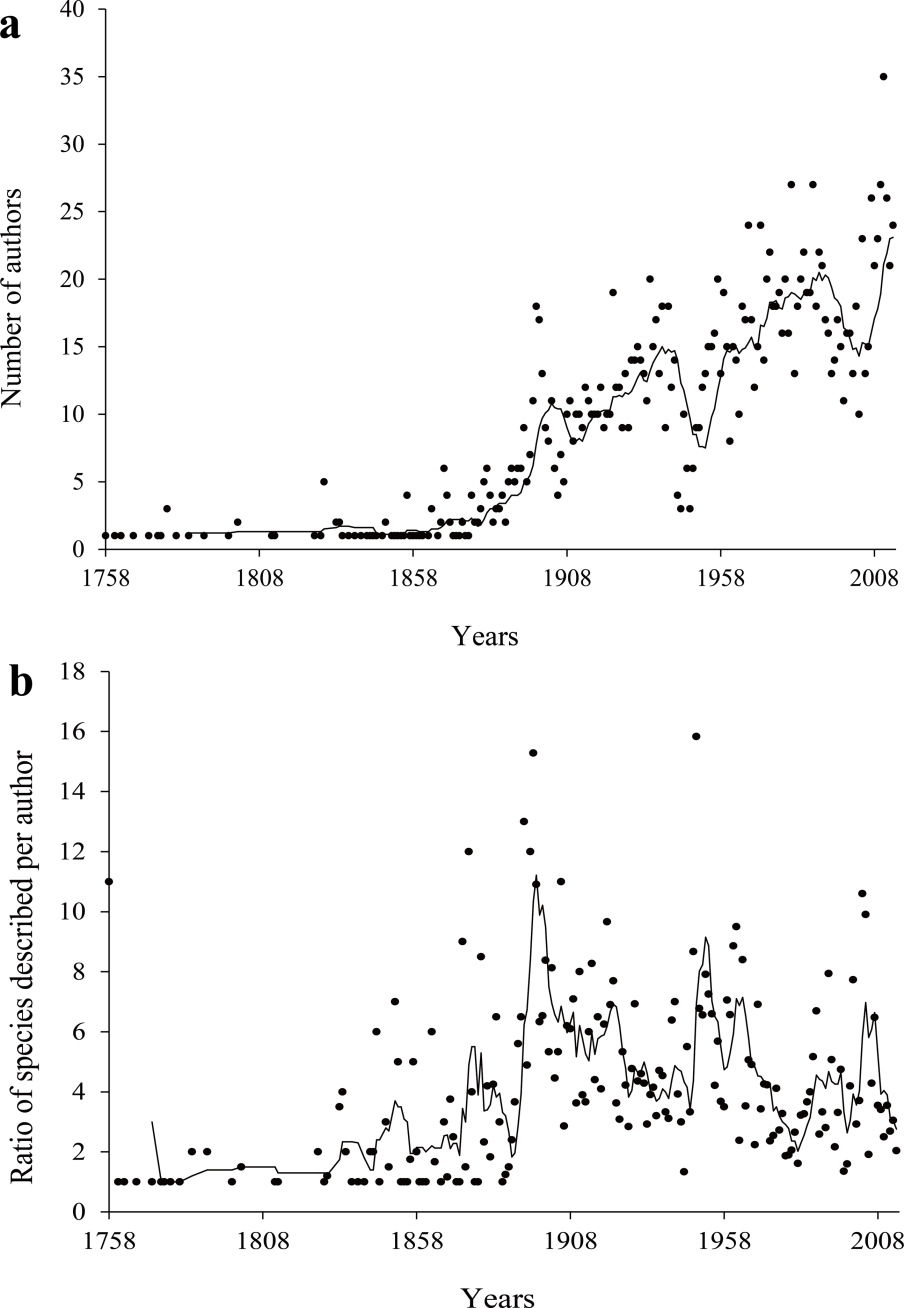
Number of authors (A) and number of species description per author over time (B). The lines plotted are a 10 year moving average.

**Figure 4:**
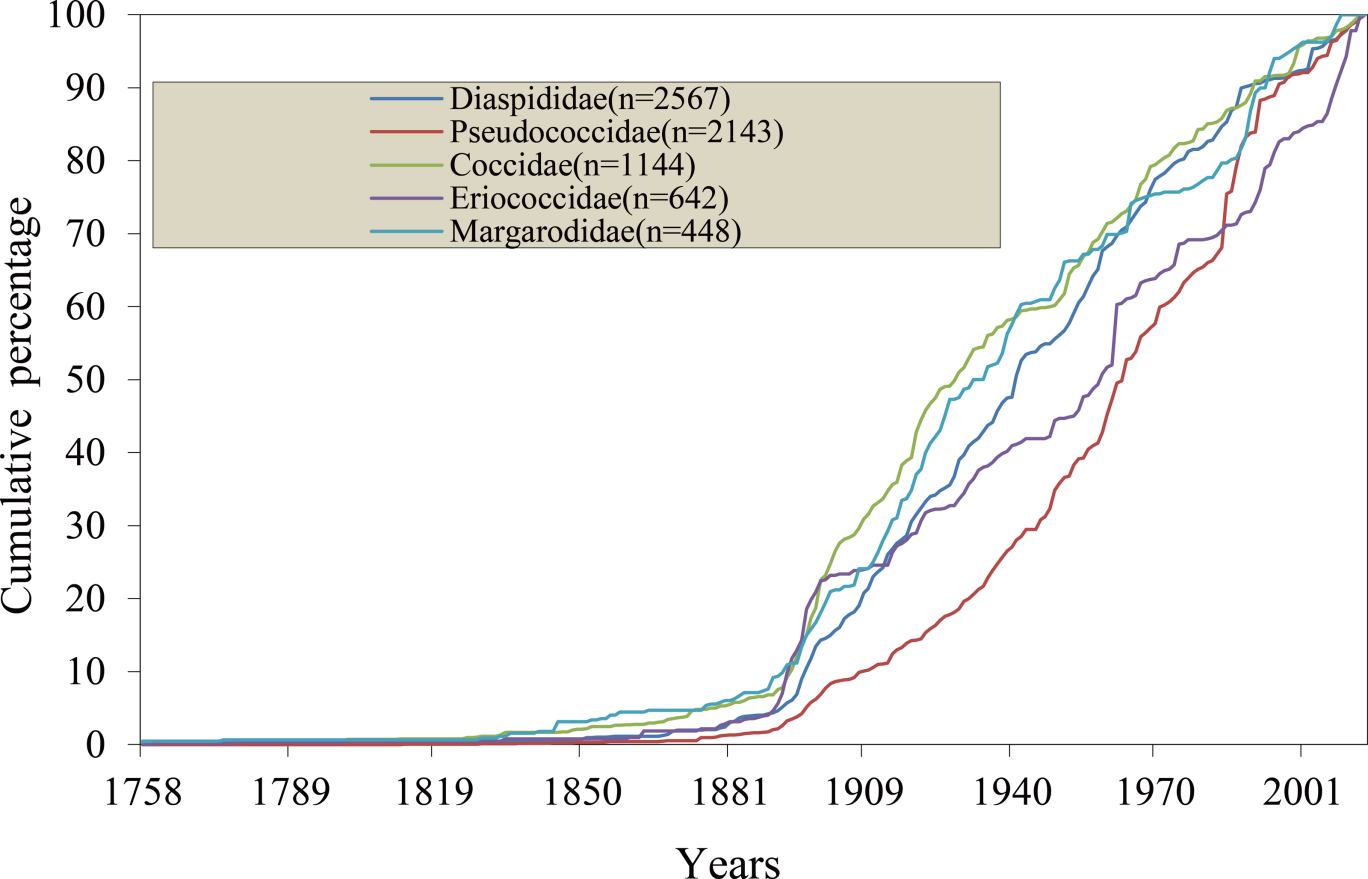
Cumulative rate of species description of the five major families from 1758 to 2014. Diaspididae, Pseudococcidae, Coccidae, Eriococcidae, and Margarodidae were showed, respectively.

The number of authors describing species increased over time (The coefficient of determination: *r*^2^ = 0.71; *P* < 0.01) (Fig. 3A). However, the number of species described per author has been decreasing since the 1960s (Fig. 3B). Among five major families, the rate of species description was variable over time (Fig. 4). Diaspididae, Coccidae and Margarodidae had similar curves of description rate, which were very low before 1900, intensely increased in 1900s, and constant until recent years. The description rate of Eriococcidae sharply increased in both the 1900s and the last decades. For Pseudococcidae, the description effort remain relatively stable after 1900, but a rapidly increase emerged in the 1980s due to a large number of new species described by Douglas J. Williams.

**Figure 5:**
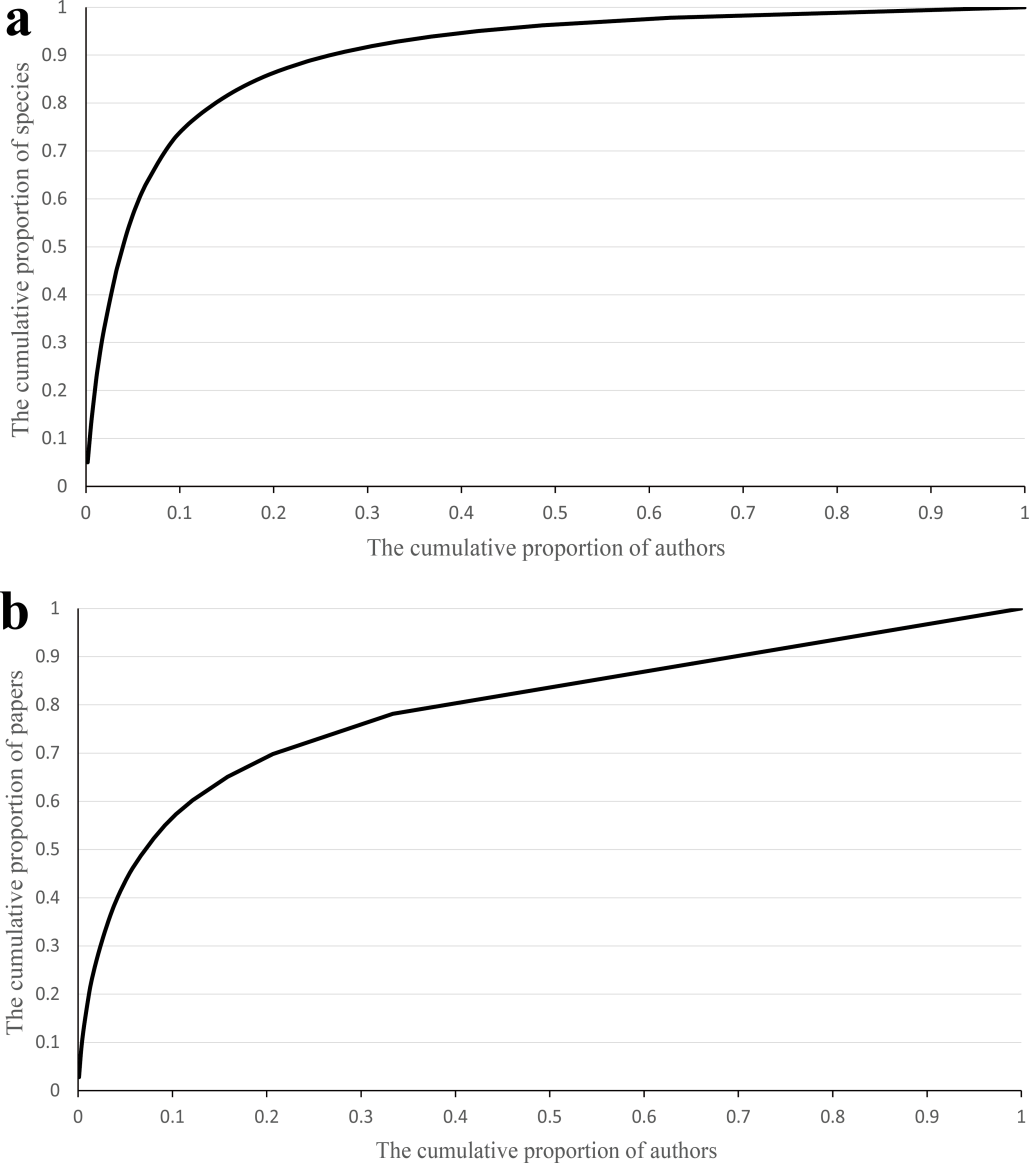
The cumulative proportion of discovered species (A) and published papers (B) against the cumulative proportion of authors.

**Figure 6:**
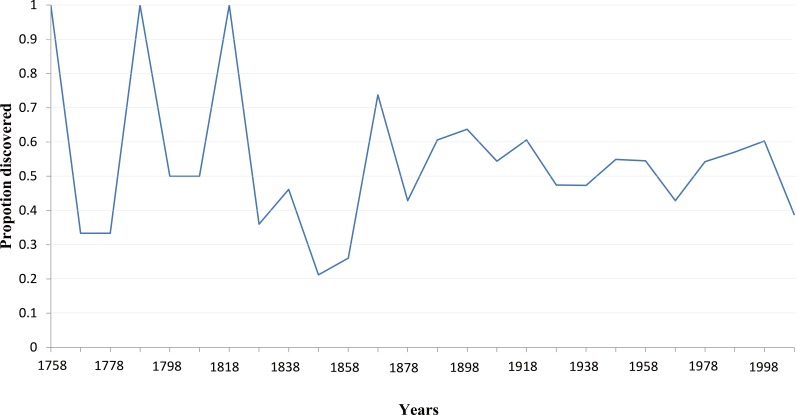
The proportion of species that the 10% most prolific authors described.

**Figure 7:**
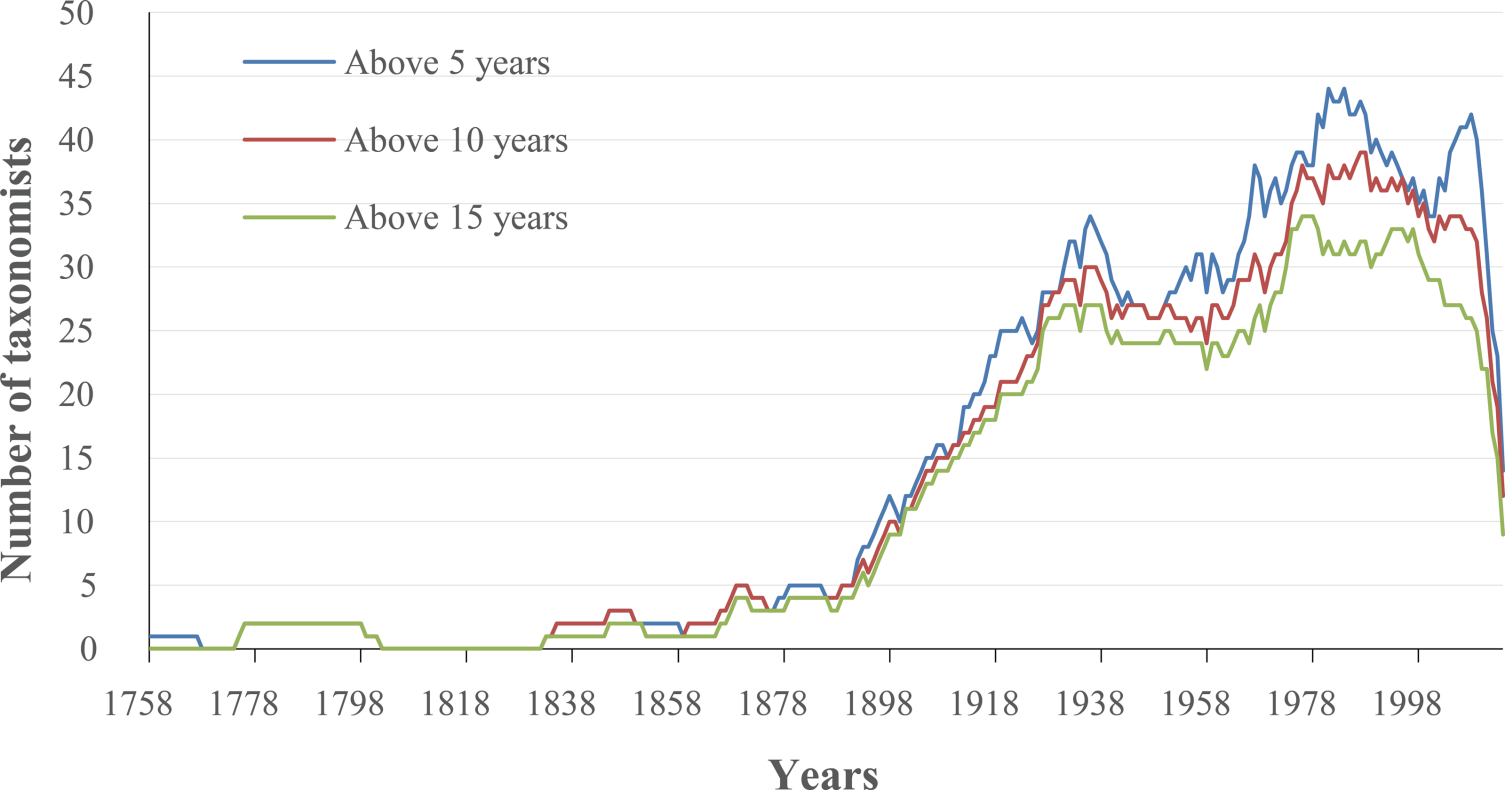
The number of taxonomists with taxonomic experience more than 5, 10 and 15 years.

**Figure 8:**
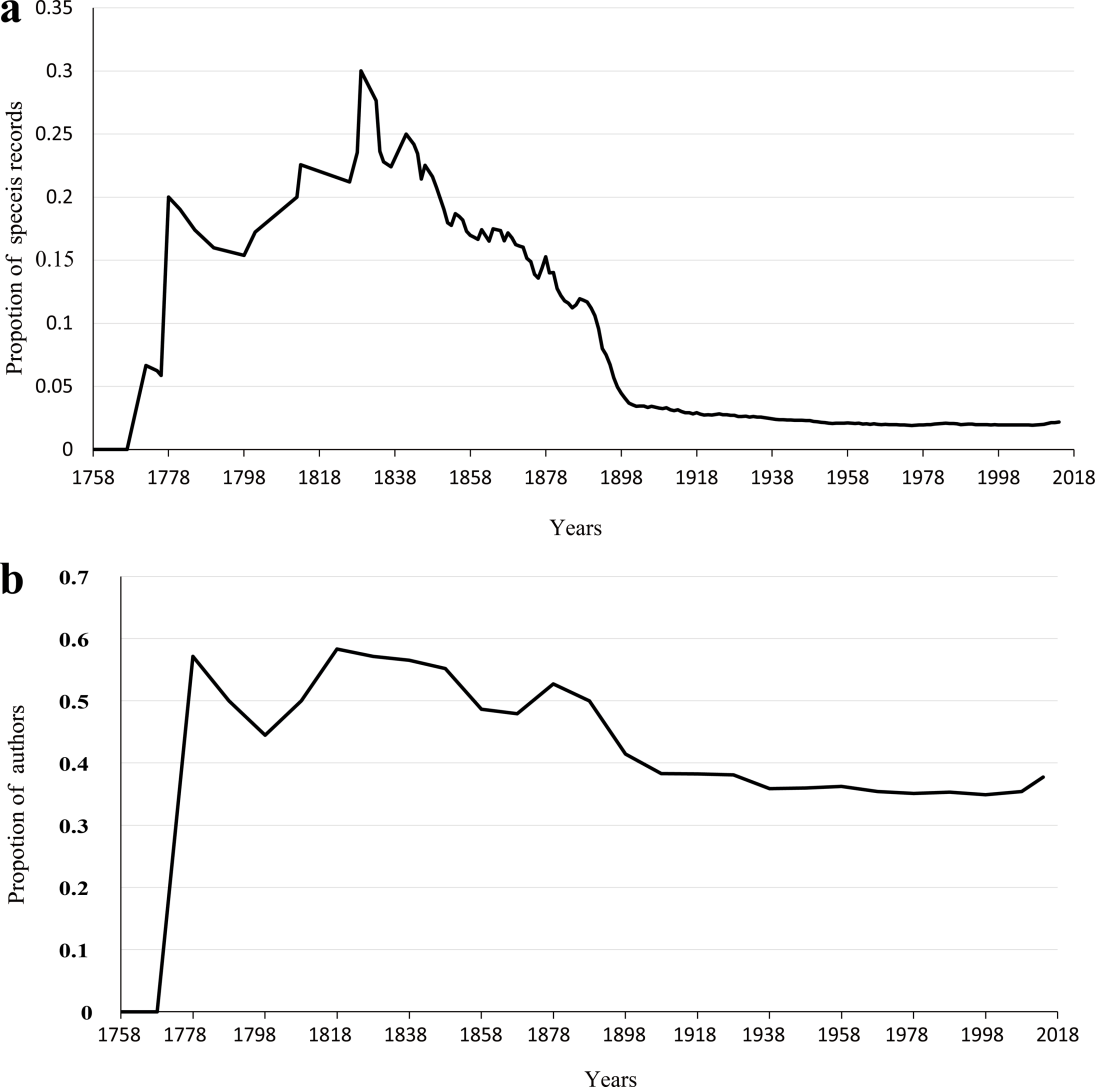
The taxonomic efforts of authors described only one species. The proportion of species discovered by authors who described only one species (A); the proportion of authors who described only one species (B).

Our analyses showed that 10% authors discovered 74% species (Fig. 5A) and published 57% papers (Fig. 5B), respectively. Furthermore, the proportion of species described by the 10% most prolific authors varied dramatically when only few authors studied on scale insect taxonomy before the 1900s, and has been relatively steady since then (Fig. 6). The trend of number of three types of taxonomists with different taxonomic experience over time were almost similar (Fig. 7): an increase since the end of nineteenth century and an obvious decrease during the Second World War. The authors who described only one species totally contributed 2% species of scale insects (Fig. 8A), and the proportion of these authors since the 1900s was about 35% (Fig. 8B).

**Figure 9:**
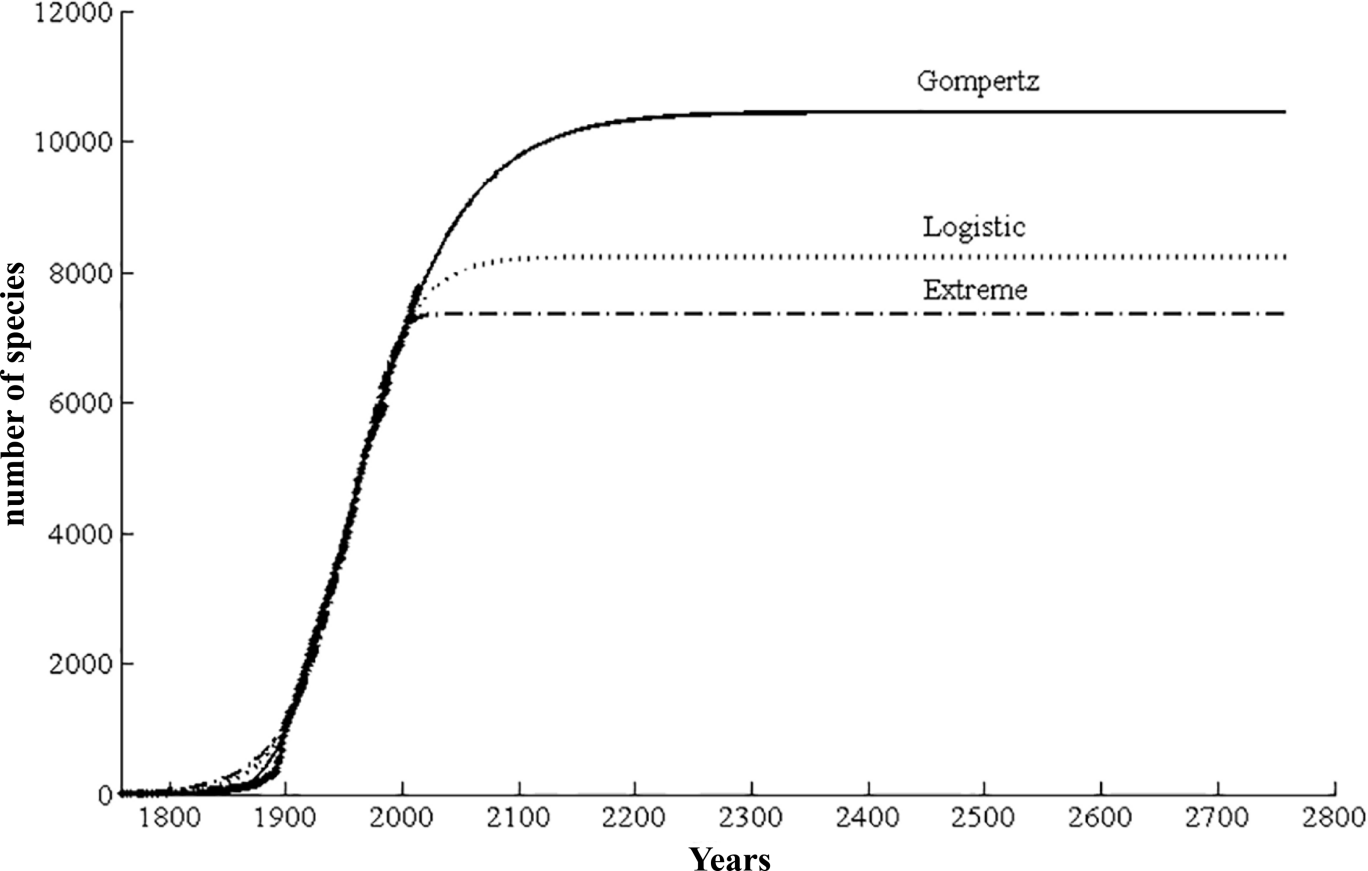
Predicted cumulative species curve for scale insects based on three curvilinear models (Extreme, Logistic, and Gompertz).

Three non-linear models showed different predicted values (Fig. 9). The Gompertz model achieved the lowest AIC value (1793.63), and had significantly better adjustment than the other two models. The best model estimated a total of 10,450 species of scale insects, of which 2,696 species (26%) remained to be described (Table 1). Meanwhile, the 95% confidence interval was narrow enough from 10,160 to 10,740.

**Table 1:** Descriptive statistics for species diversity of scale insects from 1758 to 2014 and prediction by using the curve-linear models (Gompertz, Logistic and Extreme value).

	Statistics
Number of described species	7,754
First year of description	1,758
Last year of description (up to the date of data collection)	2014
Species description rate (mean and standard deviation)	30 ± 36
AIC (Gompertz)	1793.63
AIC (Logistic)	2001.67
AIC (Extreme value)	2107.38
Number of species predicted by best model	10,450
Lower confidence limit (95%) by best model	10,160
Upper confidence limit (95%) by best model	10,740
Number of species yet to be described	2,696
Estimated year for asymptote of the best model	3,800
Percentage of undescribed species	26%

## Discussion

The average rate of species description of scale insects was very low before the year 1860, with one species per year. Identification of scale insects usually depends on mounted slides, which provide information of morphological characters with the help of microscopes. With the development of microscopes, the description rate began to increase from 1860–1886 (Fig. 1A). Some entomologists such as Signoret promoted taxonomy of scale insects at that time (Ben-Dov & Matile-Ferrero, 1995). From then on, more taxonomists such as William Miles Maskell, Theodore DruAlison Cockerell and Edward Ernest Green worked on scale insects (Kondo, Gullan & Williams, 2008), the first peak period of discovery arose in the last decade of the 19th century. The discovery rate was noticeably interrupted during the two World Wars. The year with the highest number of described species was 1985. The reason was that Douglas J. Williams published 16 papers (García et al., 2015) which described 133 of 195 species reported in that year. As Douglas J. Williams primarily focused on the morphological taxonomy of Pseudococcidae, more Pseudococcidae were discovered after 1980 (Fig. 4). The number of scale insect species described per author showed a decreasing trend in recent years (Fig. 3B). Some recent studies reported similar trend of a decline in the number of species described per taxonomist (Joppa, Roberts & Pimm, 2011; Costello, Wilson & Houlding, 2012). In consideration of the limit of total species number the Earth can support and the increase of authors, the decline of species/author ratio is understandable.

Our results indicated that a relatively small percentage of taxonomists or authors contributed high percentages of described species and papers (Fig. 5), which demonstrated that full-time professionals or taxonomists played a crucial role for the biodiversity discovery and taxonomy of scale insects. The proportion of prolific authors (Fig. 6), who were supposed to be full-time professional taxonomists, as well as the proportion of authors described only one species (Fig. 8), who were supposed to be amature taxonomists, suggest that the relative proportion of full- versus part-time taxonomists has been almost stable in the past century. Costello, Wilson & Houlding (2013) also found a similar proportion (38–42% for nonmarine species) of authors described only one species. Meanwhile, the total number of scale insect taxonomists is still increasing, which may reflect an increase of taxonomic efforts for scale insects. In recent years, some researcher may have an impression that taxonomic expertise or taxonomists have been disappearing. However, recent empirical studies indicate that taxonomists may never truly be in danger of extinction (Costello, May & Stork, 2013; Costello, Houlding & Wilson, 2014). More and more identification demands rise from other fields beyond taxonomy, such as ecology, evolution, molecular phylogeny and conservation. Therefore, more time-consuming and complex diagnostic work need be finished by taxonomists. These may lead to such an impression that taxonomists are endangered. As Costello, May & Stork (2013) pointed out, this impression is also caused by the retirement of best known taxonomists and the dilution of traditional taxonomy among new biology specializations.

The Gompertz model with lowest AIC value yielded an estimate of 10,450 scale insect species, nearly 30% of which remained to be described. The uncertainty of the extrapolation based on time–species accumulation curve has been discussed in some studies (Bebber et al., 2007; Mora et al., 2011; Costello, Wilson & Houlding, 2012). Considering the variable taxonomic effort and lack of flattening out of the cumulative description rate curve, there is considerable uncertainty on the predicted number of scale insects. Before the inventory of a taxonomic group is nearly complete, the uncertainty of the prediction using existing data still exist (Bebber et al., 2007). Our prediction needs to be interpreted with caution. Recent analyses of potential global species richness suggest that there are 2–3 million species on Earth, of which over two-thirds have already been found (Costello, Wilson & Houlding, 2012; Costello, May & Stork, 2013; Costello, 2015). In the present study, 26% of scale insects are yet to be named and remain to be discovered. This result is similar with previous studies for other groups such as 23% of marine fish (Eschmeyer et al., 2010), 25% of flowering plants (Bebber et al., 2014), 30% of sea anemones (Fautin, Malarky & Soberon, 2013) and 39% of algal (De Clerck et al., 2013). Considering the long history of Coccidology and the declining trend of species description in some major families of scale insects (Fig. 4), it is relatively reliable that about 1/3 of scale insects remain to be found and described.

A mass of work have to be done in scale insect alpha-level revisionary systematics. Same challenge also appear in other groups, 20% of the currently recognized species is considered to be undiscovered synonyms (May, 2010; Costello, May & Stork, 2013). For scale insects, some new techniques and methods based on molecular data can help solve this difficulty, including molecular identification (Deng et al., 2012; Lin et al., 2013) and endosymbiont research (Gruwell, Morse & Normark, 2007; Andersen et al., 2015). However, DNA sequences are far from enough to confirm these synonyms for scale insects. For example, among the 1,140 Coccidae species, only 41 species have sequences of the barcoding gene, mitochondria COI, in GenBank (Wang et al., 2015). With usage of new techniques and methods in taxonomy of scale insects, species diversity of scale insects can be uncovered more efficiently.

The prediction of species number can help uncover the order of magnitude of species diversity awaiting description. Furthermore, the time–species accumulation curve provides a good way to estimate the taxonomic efforts among different taxa and identify neglected taxa. To discover the one-third unknown scale insects as soon as possible in the context of increasing human impacts on ecosystem, investment in research on traditionally neglected taxa and understudied geographic regions, especially the southern hemisphere and palaeotropics (Hardy, 2013), needs to be done. Considering difficulty and complexity of identification of scale insects, efficient communication, taxonomic revisions, international collaborations, and amature taxonomist training should contribute to discovering more species of scale insects.

Our study offers insights into the discovery pattern of species diversity and taxonomic efforts for scale insects. Considering the threats of species extinctions, taxonomic research has never been so urgently required (Costello, 2015). We need to increase investment in research on species diversity of scale insects, as well as accelerate taxonomic efforts for this important insect group.

##  Supplemental Information

10.7717/peerj.2526/supp-1 Table S1 The final dataset including a total of 7754 species of extant scale insects

## Additional Information and Declarations

### Data Availability

The following information was supplied regarding data availability: The raw data has been supplied as Supplemental File.
